# Renovating Old Oil Paintings

**Published:** 1884-11

**Authors:** 


### RENOVATING OLD OIL PAINTINGS.

IN cleansing old paintings that have become dingy with soot and coal dust, substances are frequently employed that injure the painting by acting on the lighter and more delicate tints and shades.

Von Bibra has discovered a method which, according to Weick's *Gewerbe Zeitung*, is both safe and rapid.

The painting is first removed from the frame, and the dust and smoke brushed off with a pencil or feather. After this it is washed with a sponge dipped in well water. It is next covered with a thick layer of soap ; shaving soap is the best for the purpose, because it remains moist and does not dry on. After the soap has been on eight or ten minutes i is all washed off with a strong brush or pencil, adding a little water if necessary. The soap that still adheres is rinsed off sufficiently with water, and the picture left to dry.

When completely dry it is further cleansed with nitrobenzol. This chemical preparation is also known as nitrobenzine, artificial oil of bitter almonds, essence of mirbane, and is a yellowish, oily (*very poisonous*) liquid, with a powerful smell of bitter almonds. It is formed when coal-tar benzol is mixed with fuming or concentrated nitric acid under suitable precautions. The nitro-benzol is poured into a dish or soup plate, and a clean linen rag dipped in it, and passed over the painting. This quickly removes all the adherent dirt. This linen rag must be frequently exchanged for a clean one. When the rag remains clean after going over it repeatedly, the cleansing is finished.

If the colors look dull after going over it the last time and letting it dry, it is given a thin coat of the finest olive oil, and after a while must be varnished with a good, quickly drying varnish.

It is claimed that the dirtiest oil paintings, when cleansed as above described, acquire their original colors and freshness.

